# Wetting of a Hydrophobic Surface: Far-IR Action Spectroscopy and Dynamics of Microhydrated Naphthalene

**DOI:** 10.1021/acs.jpclett.3c02854

**Published:** 2023-11-28

**Authors:** Alexander K. Lemmens, Piero Ferrari, Donatella Loru, Gayatri Batra, Amanda L. Steber, Britta Redlich, Melanie Schnell, Bruno Martinez-Haya

**Affiliations:** 1 Chemical Science Division, Lawrence Berkeley National Laboratory, Berkeley, California 94720, United States; 2 Radboud University, Institute of Molecules and Materials, HFML-FELIX, Toernooiveld 7, 6525 ED Nijmegen, The Netherlands; 3 Deutsches Elektronen-Synchrotron DESY, Notkestr. 85, 22607 Hamburg, Germany; 4 Department of Physical and Inorganic Chemistry, Faculty of Science, University of Valladolid, 47011 Valladolid, Spain; 5 Institut für Physikalische Chemie, Christian-Albrechts-Universität zu Kiel, Max-Eyth-Str. 1, 24118 Kiel, Germany; 6 Center for Nanoscience and Sustainable Technologies (CNATS), Department of Physical, Chemical and Natural Systems, Universidad Pablo de Olavide, 41013 Seville, Spain

## Abstract

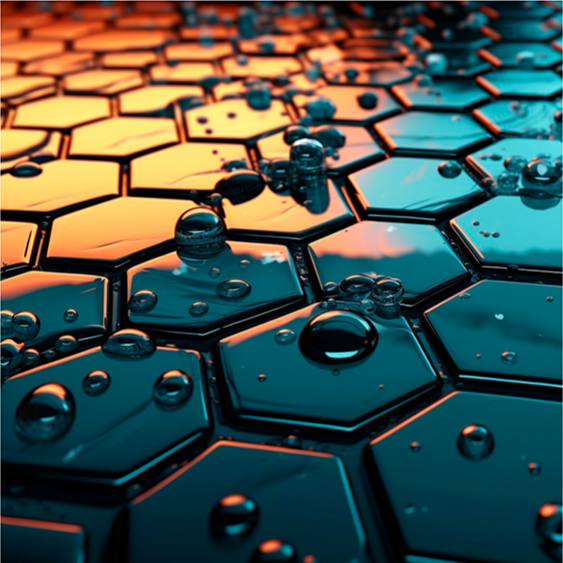

The interaction of water and polycyclic aromatic hydrocarbons is of fundamental importance in areas as diverse as materials science and atmospheric and interstellar chemistry. The interplay between hydrogen bonding and dipole−π interactions results in subtle dynamics that are challenging to describe from first principles. Here, we employ far-IR action vibrational spectroscopy with the infrared free-electron laser FELIX to investigate naphthalene with one to three water molecules. We observe diffuse bands associated with intermolecular vibrational modes that serve as direct probes of the loose binding of water to the naphthalene surface. These signatures are poorly reproduced by static DFT or Møller–Plesset computations. Instead, a rationalization is achieved through Born–Oppenheimer Molecular Dynamics simulations, revealing the active mobility of water over the surface, even at low temperatures. Therefore, our work provides direct insights into the wetting interactions associated with shallow potential energy surfaces while simultaneously demonstrating a solid experimental–computational framework for their investigation.

Hydrophobicity results from the often subtle interplay of water–water, solute–solute, and water–solute or water–substrate interactions. It is a major player in determining the properties of bulk materials and a key factor in supramolecular chemistry and biochemistry.^1,2^ Hydrophobicity is, for instance, crucial in processes involving molecular recognition, macromolecular folding, or reversible binding of water to biomolecular or catalytic active sites.^3−5^ The weak interactions between water and polycyclic aromatic hydrocarbons (PAHs), addressed in this investigation, typically result in their insolubility. Nevertheless, soot or carbonaceous particles play a relevant role in the nucleation of water in the atmosphere^6−8^ and in interstellar environments.^9,10^ First-principle approaches have often adopted microhydration strategies to understand water–solvent versus water–water interactions, in which the solvation shell of a given substrate is scrutinized in isolated clusters with a well-defined number of water molecules.^1,2,11−17^ IR spectroscopy has been demonstrated to be an excellent tool for investigating interactions in water–PAH clusters and related systems,^12,13,15,16,18^ along with complementary work in the microwave^17,19−21^ and (V)UV^22,23^ spectral regions. Apart from structural elucidation, those studies have exposed the subtle balance between the strong hydrogen bonding within the water network and the weaker dipole−π interactions between water and the PAH substrate.

In this work, far-infrared spectroscopy is employed to investigate the vibrational signatures of isolated complexes of naphthalene (henceforth, N) with up to three water (W) molecules (complexes will be denoted as NW1–NW3). The far-IR spectral features of water clusters and hydrated PAHs have received considerable attention, largely motivated by their contribution to IR absorption in the Earth’s atmosphere.^6,7,24−27^ Previous spectroscopy studies of naphthalene microhydration suggest that water molecules may dock over the aromatic plane through OH−π interactions^13^ or more peripherally through CH–O interactions,^28,29^ with recent microwave spectroscopy experiments on phenanthrene–water clusters highlighting the importance of quantum tunneling in the movement of water across the surface of PAHs.^20^ Interestingly, remarkable charge-induced structural changes have been observed in these systems. For instance, in the anionic form of the NW complex (as in the neutral form) the water molecule is most stable on top of the PAH plane,^13,15^ while the cationic form favors a peripheral docking of the water molecule through CH^δ+^···OH interactions.^29,30^ The structure of NW complexes is also largely affected by protonation, with the proton being retained by naphthalene for single hydration and by the water moiety for larger clusters.^12,31^ The investigation of electronically excited states and photoionization of NW complexes has also provided valuable information in this context.^22,32^

The previous vibrational infrared studies of NW clusters have typically focused on the C-H and O-H stretching regions, which indirectly reveal information on the noncovalent water–PAH interactions. Instead, the far-IR light employed in this study probes intermolecular vibrational modes and, therefore, serves as a more direct probe of the naphthalene–water potential energy surface,^33,34^ also allowing for an investigation of the perturbing effect of the PAH substrate on the structure and vibrational features of the water clusters. Moreover, previous spectroscopic experiments on microhydrated naphthalene have been mostly interpreted in terms of static computations, e.g. employing Density Functional Theory (DFT), Møller–Plesset (MP2), or Coupled-Cluster approaches.^15,35^ The naphthalene–water potential energy surface is, however, very shallow, with perturbations to the electronic structure of naphthalene inducing significant changes in the geometry of the complexes. At the low but finite temperatures reached in molecular beams, shallow potentials go hand in hand with fluxional geometries, which warrants far-IR spectral analysis to be performed within a dynamic, rather than a static framework. Here, we show that Born–Oppenheimer molecular dynamics (BOMD) simulations provide a suitable formalism to describe weakly bound PAH–water clusters in molecular beams.^36−39^ The spectral features observed experimentally are interpreted from the BOMD computations in terms of an efficient diffusion of the water moiety on the “hydrophobic” substrate, leading to a dynamic sampling of qualitatively different coordination arrangements. Also the BOMD approach intrinsically accounts for anharmonicity, which has been pointed out as a challenging factor in the spectral analysis of PAH–water clusters.^16^

The resonance-enhanced multiphoton ionization (REMPI) spectra of the naphthalene monomer and of its microhydrated complexes are shown in Figure 1(a). A typical time-of-flight mass spectrum is given in Figure S1 of the Supporting Information (SI), to illustrate the distribution of cluster sizes observed in the molecular beam. Because the weakly bound NW clusters produced in the experiment can fragment upon photoexcitation, care must be taken when interpreting the recorded mass-selected REMPI or IR-action spectra. Sharp absorption lines in the REMPI spectrum of the naphthalene monomer (lower black trace in Figure 1(a)) are accompanied by broader absorption features that can be ascribed to the naphthalene dimer dissociating upon absorption of one or multiple photons. Similar dissociative contributions from heavier clusters apply to the REMPI spectra of the NW1–3 complexes. Fortunately, the clear separation of the electronic transitions of the NW1–3 complexes allows us to neatly record their individual IR spectra. We do not focus on larger complexes, because of their low S/N ratio and overlap in REMPI transitions. In Figure 1(a), the black vertical dotted lines indicate transitions corresponding to N. The transitions that are red-shifted by about 40 cm^–1^ from these lines are the result of the NW2 complex losing one water and ending up in the NW1 *m*/*z* channel. This can be appreciated when comparing the NW1 and NW2 REMPI spectra, as the peaks line up. Similarly, the peaks blue-shifted by about 55 cm^–1^ from the UV1 transition in both the NW1 and NW2 REMPI spectra are attributed to the NW3 fragmenting and ending up in the NW1 and NW2 *m*/*z* channels. The broadening of the NW1–5 peaks may originate from either dynamics or less efficient cooling of the weakly bound clusters. In general, the vibronic bands show the same behavior with similar red- or blue-shifts depending on the different hydration levels. One striking difference is the lack of intensity observed around the origin transition (32032 cm^–1^) of naphthalene upon hydration. Similarly, no intensity is observed around the band at 33019 cm^–1^ previously assigned to 7_0_^1^.^51^ We speculate that the mixing of electronic states is affected by the complexation of water, resulting in a loss of intensity for some transitions. The transitions used in our experiments are labeled in Figure 1(a) as UV1–3 and NW1–3, respectively.

**Figure 1:**
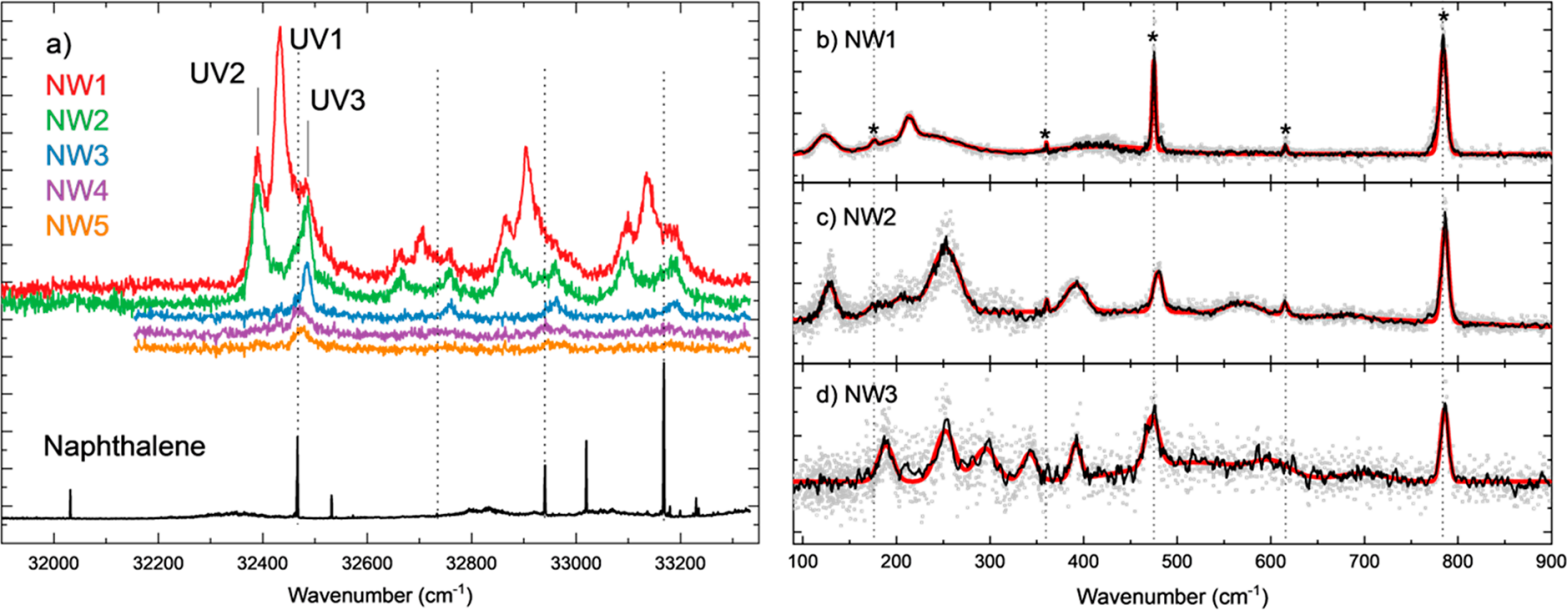
(a) 1 + 1′ REMPI spectra of naphthalene–water_1–5_ (NW1–5) complexes (colored traces). The curves are displaced in the *y*-axis for visualization purposes and are ordered as listed in the legend. The lower REMPI spectrum (black trace) is recorded at the mass of the bare naphthalene molecule; the broader contributions in this spectrum are assigned to dimer fragmenting in the monomer *m*/*z* channel. Similar photoinduced fragmentation is observed for the NW1–5 complexes, losing one or more water molecules. The electronic transitions used in the ion-dip measurements are labeled UV1–3 for NW1–3, respectively. (b-d) Far-IR gas phase ion-dip action spectra of jet-cooled NW1–3 complexes in black, averaged over three IR scans of 30 averages per wavenumber each (gray dots). The vertical black dotted lines and asterisks indicate peaks originating from the naphthalene moiety. The red traces result from a sum of Gaussian fits to the experimental bands (see Table S1)

The far-IR ion-dip spectra of the NW1–3 water complexes are represented in panels (b–d) of Figure 1. An array of sharp and broad bands is observed in each of the spectra. The sharper bands, marked with asterisks in Figure 1(b), can be assigned to the vibrational modes of naphthalene.^52^ The particularly intense band at 785 cm^–1^ is associated with the collective out-of-plane bending vibration of the peripheral C-H groups, while bands of varying intensity are also observed at 615 cm^–1^ (asymmetric CCC bending in the two naphthalene rings), at 475 and 360 cm^–1^ (out-of-plane and in-plane CCC bending of the central carbons of naphthalene, respectively), and at 170 cm^–1^ (breathing deformation of naphthalene). The positions of these bands are weakly dependent on the level of hydration of naphthalene. Only the band at 475 cm^–1^ displays a sizable shift in the NW2 complex with respect to the NW1 and NW3 complexes. This suggests a differentiated interaction of the dimer with the central CCC moiety, which would be consistent with the computational predictions, as discussed below.

The most notable spectral features induced by the hydration of naphthalene are the diffuse band structures observed in the 100–500 cm^–1^ range for the NW1 complex and extending up to 800 cm^–1^ for the NW2 and NW3 complexes. Note that the IR laser bandwidth scales with energy (typically 0.5% of the central wavenumber), so that if peaks were laser-bandwidth limited, they would be narrower at lower wavenumbers. The observed diffuse bands are partly related to the intermolecular vibrational modes of the naphthalene–water complex, for the NW1 complex exclusively so. The computations for NW1 predict bending motions (rocking and wagging) of water coupled to breathing deformations of the naphthalene backbone. The energies of these modes are closely related to the water–naphthalene coordination arrangement. Hence, the diffuse nature of the observed bands suggests a loose binding of the water molecule and a dynamically changing binding configuration. The BOMD computations described below support the idea that the band broadenings are determined by intermolecular dynamics. Alternatively, coupling to the dissociation coordinate may also result in broadening,^53^ although this does not appear to constitute a relevant contribution here. The spectra of the NW2 and NW3 complexes display a larger number of band components, partly due to the internal modes of the H-bonded arrangements of the water dimer and trimer. Interestingly, the band features in the spectra become progressively less broadened in the NW2 and NW3 clusters than observed for NW1 below 500 cm^–1^. This can be more quantitatively appreciated from the cumulative Gaussian fit of the spectra described in Table S1 of the SI, leading to the red traces in Figure 1(b–d). The reduced broadening suggests a more rigid configuration of the water dimer and trimer on the naphthalene surface in comparison to the single water complex, also supported by the microwave spectroscopy work, which shows larger clusters to be more ‘locked’ in place.^17,19−21^

A general description of the conformational landscape of the NW1–NW3 complexes seems suitable as a preliminary step toward an in-depth rationalization of the recorded far-IR spectra. Relevant low-energy configurations are included in Figures 2–4. Figure S2 of the SI also includes the relative energies of the conformers at the different levels of theory considered in this study. In the most stable configuration of the NW1 complex (denoted as NW1-1), the water molecule docks on the central part of naphthalene. Incidentally, water is slightly displaced from the center in the DFT computations, irrespective of the functional employed, while the conformation becomes C_2v_ symmetric at the MP2 level. The peripheral docking through CH···O H-bonding interactions results in higher energies at all computational levels (conformations NW1-2 and NW1-3). The potential energy surface associated with the naphthalene–water interactions in the singly hydrated complex is described in Figure S3, in terms of cross sections along orthogonal directions over the naphthalene plane and around the naphthalene rim. In the NW2 and NW3 complexes, the water molecules cluster in configurations that resemble those of the isolated dimer^37^ and trimer,^24^ with some distortion to optimize coordination with the N substrate. Any configuration associated with individual water molecules adsorbed on different domains of the aromatic substrate was found to lie higher in energy and/or to be dynamically unstable. For NW2, two main types of low-energy configurations are devised: in the NW2-1 and NW2-3 conformers, one water molecule of the dimer interacts with peripheral CH groups, while in the NW2-2 conformer, the dimer sits on top of naphthalene, forming a two-fold coordination with the π cloud of the aromatic moiety. Conformation NW2-3 turns out to be unstable at all of the levels of theory, typically converging to conformation NW2-1 or to higher lying configurations upon optimization. A similar arrangement to NW2-1 was observed in the phenanthrene–water complex.^21^ Imposing symmetry constraints to preclude conformational migration in NW2-3 resulted in one negative frequency associated with a torsional mode of the water dimer. Alternative configurations, based on other structural characteristic of the water dimer,^54^ are unlikely to have a large contribution, since they would not be able to coordinate to naphthalene with two anchor sites as strong as the ones present in NW2-1 and NW2-2. Interestingly, the relative energies of the NW2-1 and NW2-2 conformers are dependent on the level of theory employed, as well as on the choice of zero-point corrected energies or of free energies as reference (see Figure S2). Therefore, it is inconclusive which configuration is more stable. Finally, in the NW3 complex, different relative orientations of the water molecules in the trimer with respect to naphthalene are possible, leading to different combinations and amounts of OH···π and CH···O interactions with naphthalene, which have all been considered. In the most stable conformation, NW3-1, the water cluster adopts a configuration close to that of the free trimer, whereas NW3-2 and NW3-3 involve relative reorientations of one of the water molecules. These latter conformations lie higher in energy than NW3-1, with NW3-2 being unstable at some levels of theory.

**Figure 2:**
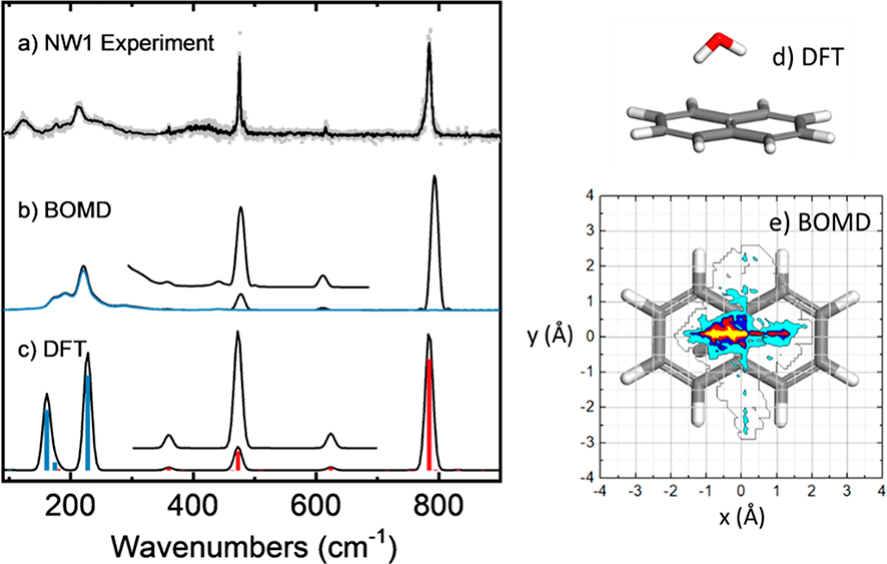
(a) Far-IR gas phase ion dip action spectra of jet-cooled naphthalene–water (NW1) complexes (same as in Figure 1(b)) compared to the predictions of the (b) BOMD and (c) harmonic DFT (B3LYP) computations. The insets in parts (b) and (c) are the result of a 5× multiplication and a *y*-axis offset. Computational frequencies are scaled by a factor of 0.98. The blue trace in the BOMD spectrum is obtained from the deconvolution of the water motions within the complex, whereas the corresponding harmonic modes in the DFT spectrum involving water are represented as blue bars (red bars denote pure naphthalene modes). (d) Lowest energy conformation of the NW1 complex at the B3LYP level. (e) Spatial distribution of the O atom of the water molecule projected on the naphthalene plane, as derived from the BOMD trajectory (yellow corresponds to highest probability).

**Figure 3:**
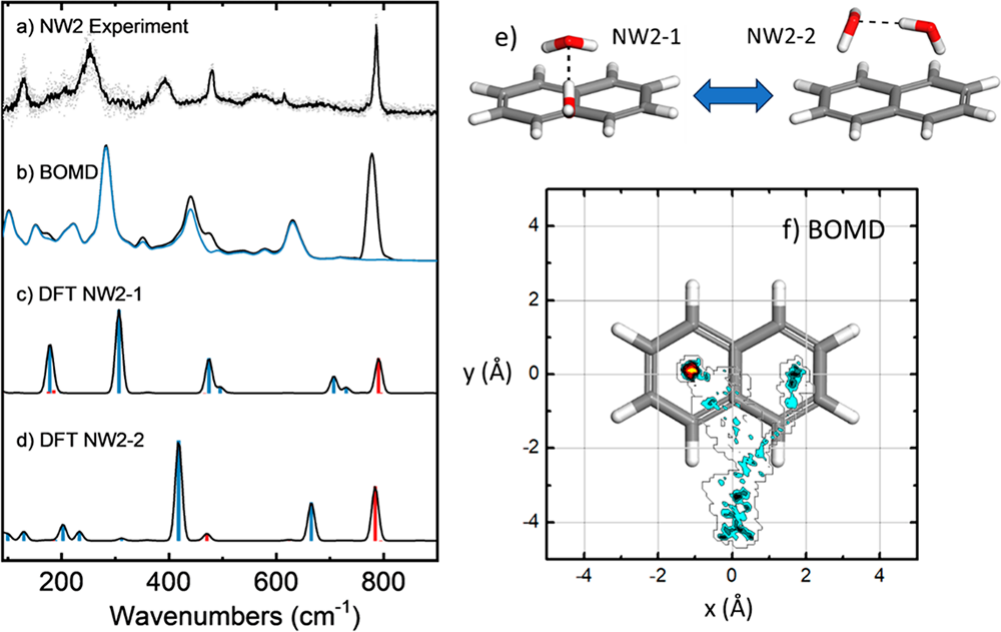
(a) Far-IR gas phase ion-dip action spectra of jet-cooled naphthalene–water_2_ (NW2) complexes compared to the predictions of the (b) BOMD and (c,d) harmonic DFT (B3LYP) computations; computational frequencies are scaled with a factor of 0.98. The blue trace in the BOMD spectrum is obtained from the deconvolution of the water motions within the complex, whereas the corresponding harmonic modes in the DFT spectrum involving water are represented as blue bars (red bars denote pure naphthalene modes). (e) The two conformations of lowest energy of the NW2 complex at the B3LYP level, and (f) spatial distribution of the O atom of the water molecules projected on the naphthalene plane, as derived from the BOMD trajectory.

**Figure 4:**
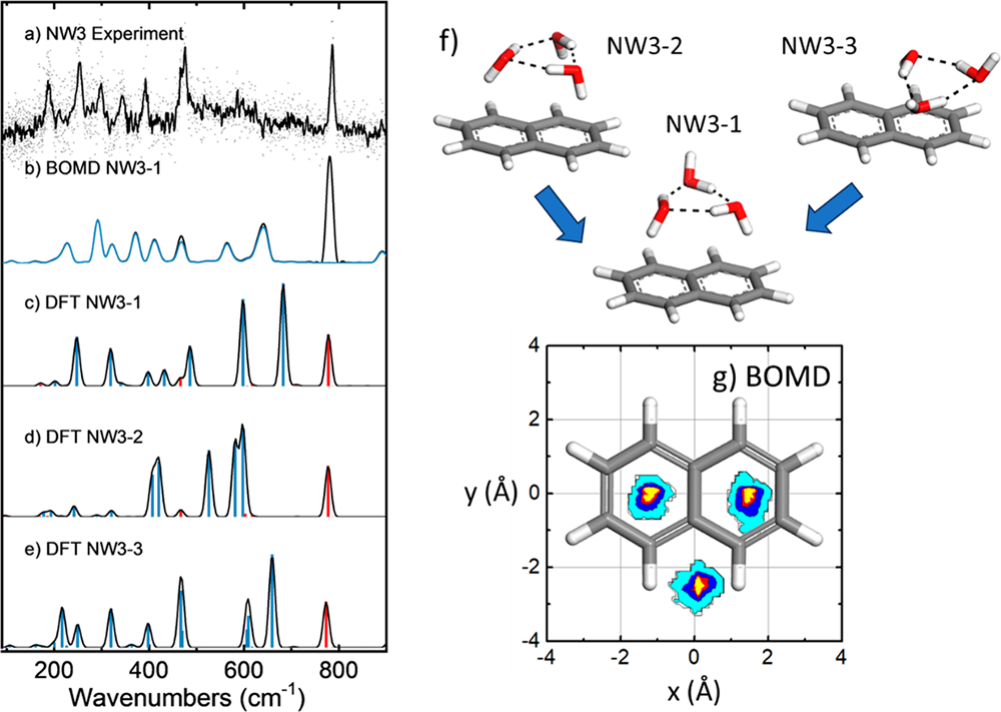
(a) Far-IR gas phase IR-UV ion-dip action spectra of jet-cooled naphthalene–water_3_ (NW3) complexes, compared to the predictions of the (b) BOMD and (c–e) harmonic DFT (B3LYP) computations; computational frequencies are scaled with a factor 0.98. The blue trace in the BOMD spectrum is obtained from the deconvolution of the water motions within the complex, whereas the corresponding harmonic modes in the DFT spectrum involving water are represented as blue bars (red bars denote pure naphthalene modes). (f) Lowest energy conformations of the NW3 complex, and (g) corresponding spatial distributions of the O atom of the water molecules projected on the naphthalene plane, as derived from the BOMD trajectory.

The conformations described are energy minima on the potential energy surface of the complexes, constituting valuable references to rationalize the structure of hydrated naphthalene. It will be argued that the far-IR spectra of the NW1–3 complexes can only be accurately understood in terms of dynamic structures, eventually connecting different low-energy configurations such as the ones just described. In the following, the main spectral features observed for each of the systems are discussed.

The experimental far-IR spectrum of the NW1 complex is compared to the predictions of DFT and BOMD computations in Figure 2. Static calculations, such as DFT or MP2, provide the frequencies of the fundamental vibrational modes of an equilibrium configuration of the system. Harmonic frequencies, optionally with anharmonic corrections, may then be compared with the experimental bands. In doing this, empirical broadenings are occasionally introduced mainly to resemble the laser bandwidth and the rotational envelopes of the bands observed experimentally (a 15 cm^–1^ full width at half maximum Gaussian broadening has been employed throughout this study). This approach fairly accurately describes the vibrational modes of the rigid naphthalene monomer (red bars in Figure 2(c)) but does not suffice to explain the broader bands associated with the intermolecular vibrational modes involving water motions. Figure 2 makes clear that DFT predicts the presence of intermolecular water–naphthalene vibrational modes in the low-energy flank of the spectrum, in line with the experiment (>300 cm^–1^, blue bars) but fails to explain the diffuse nature of the vibrational bands. Figure S5 includes predicted spectra produced with the M06-2x and ωB97xD functionals and with MP2. Whereas the predicted position of low-energy bands is sensitive to the computational method, none of them improves the comparison with the experiment with respect to B3LYP-D3. In addition, the contrast between the harmonic and anharmonic DFT calculations, also shown in Figure S3, suggests a major influence of anharmonicity on the far-IR region of the spectrum of the NW1–3 clusters.

In the BOMD calculations, anharmonicity is intrinsically taken into account, as the motion of the water molecule within the complex proceeds on an inherently anharmonic intermolecular potential energy surface. Calculating the IR spectrum from the BOMD trajectories relies on the time evolution of the dipole moment on that PES. The weak water–naphthalene interactions, combined with the finite experimental temperatures, make it likely that the complex is not limited to the PES minimum, in contrast to static computations that are essentially representing a formal 0 K picture. Figure S3 shows that the potential energy surface is indeed quite shallow and can be expected to promote an active translational and orientational freedom of the water molecule. In this scenario, BOMD computations can be foreseen to be a more suitable framework to capture the fluxional complexation of water and the associated diffuse vibrational signatures. Figure 2(b) shows that the BOMD computation for the NW1 complex indeed reproduces the broad-band signatures associated with modes involving water bending motions. In particular, in the 100–300 cm^–1^ spectral region, the BOMD predicts a broadened overlap between the bands associated with the rocking and wagging modes of water with respect to naphthalene. The deconvolution of the water motions alone leads to the blue trace depicted on top of the BOMD spectrum in Figure 2(b), which supports this assignment. Moreover, the positions of the pure naphthalene vibrational bands are also in close agreement with the experiment (and with the DFT computation). We note, however, that the BOMD peak widths are somewhat larger than in the experiment and the two lowest frequency peaks have weaker intensities compared to the peaks originating from the water complexation. Nevertheless, it is clear that the BOMD computation captures the key structural and IR spectral features of the NW1 complex. The mobility of water in the NW1 complex, as inferred for the BOMD trajectory, is visualized in Figure 2(e), by plotting the 2D distribution of the O atom projected on the plane of the naphthalene substrate. It can be observed that water undergoes transverse motions along the long axis of the aromatic structure and, to a lesser extent, also along the short axis. Such motions are consistent with the topology of the PES and account for the spectral broadening of the vibrational bending modes associated with the water–naphthalene coordination, which are directly affected by the dynamic behavior of the adsorbed water molecule. The asymmetry of the spatial distribution in the current 100 ps long trajectory should disappear in the limit of long simulation times, but it should be noted that the infrared spectrum is unlikely to change significantly with longer runs.

The far-IR spectrum of NW2 is compared with computational predictions in Figure 3. The analysis of the DFT-B3LYP-D3 spectra for the NW2-1 and NW2-2 prototypical conformers allows for the assignment of most of the observed bands (see Figure S5 for the qualitatively similar M06-2x, ωB97xD, and MP2 spectra). The most pronounced contribution from pure naphthalene modes is again the narrow peak at 790 cm^–1^ (CH out-of-plane bending, red bars), whereas at lower energy, the naphthalene vibrational transitions are weaker or mixed with bending modes of the water moiety (blue bars). The bands within 670–750 cm^–1^ in the two conformers are associated with an internal mode of the water dimer, namely bending of the O-H···O bond angle. The two bands observed experimentally in the 300–500 cm^–1^ range arise from concerted wagging and rocking motions of the water molecules in the dimer. In conformer NW2-1, this mode couples with naphthalene breathing motions, which accounts for the band observed at 475 cm^–1^, whereas the same mode for NW2-2 is red-shifted and gives rise to the somewhat broader band observed at 375 cm^–1^. Finally, lower energy vibrational modes around 200 cm^–1^ are associated with wagging and torsional bending modes of the water dimer relative to naphthalene. These transitions are broadened in the experimental spectrum, presumably as a consequence of the dynamic coordination of the water dimer with the naphthalene substrate, in a qualitatively similar way as described above for the NW1 complex.

Figure 3 shows that the BOMD computation resembles the band broadenings in the NW2 spectrum, qualitatively describing the experimentally observed diffuse bands, despite some shifts of up to ∼50 cm^–1^ in the precise band positions. The detailed analysis of the BOMD trajectory reveals that thermal fluctuations dynamically connect different types of coordination arrangements of naphthalene to the water dimer. Namely, the contour diagram (Figure 3(f)) describing the positions of the O atoms with respect to naphthalene shows that the dimer diffuses over the aromatic plane, adopting configurations of the NW2-1 type, with eventually one water molecule docking on the rim and hence sampling configurations of the NW2-2 type. Single point energies along an excerpt of the BOMD trajectory where the NW2-2 transitions to the NW2-1 are shown in Figure S4, giving an illustration of the barrier that was overcome on this run. The NW2-3 configuration described above was found to be dynamically unstable and rarely sampled, even when it was used to seed the BOMD computation. The dynamic averaging of the overall configurations during the BOMD trajectory results in the IR spectrum shown in Figure 3(b). The fair correspondence of the vibrational band structures derived from the BOMD computation with the experiment strongly supports the dynamic nature of the complex, resulting from the loose binding of the water dimer to naphthalene. However, complexation reduces the degree of freedom of the water moieties with respect to the isolated water dimer. For instance, the hydrogen bond exchange in the bare water dimer,^55^ predicted in the BOMD simulations we performed under the same conditions, is inhibited upon complexation with naphthalene.

The experimental far-IR spectrum of NW3 is compared with computations in Figure 4. The spectrum features a larger number of bands with narrower envelopes than those of its NW1 and NW2 counterparts, which in light of the computations follows from the increased number of vibrational modes in the water trimer and its greater rigidity. The IR spectrum displays the characteristic vibrational naphthalene CH bending transition at 786 cm^–1^, followed by internal bending modes of the H-bonding network of the trimer between 480 and 750 cm^–1^. At lower wavenumbers (<450 cm^–1^), the rocking and torsional modes of the water trimer–naphthalene coordinating bonds are observed.

According to the three prototypical low-energy types of anchoring of the water trimer to naphthalene described above (conformers NW3-1 (most stable), NW3-2, and NW3-3), one- to three-fold OH−π interactions may be present, along with CH···O peripheral interactions for the water molecules with OH bonds oriented away from naphthalene. Conformer NW3-2 (three-fold OH−π anchoring) is unstable at some levels of theory (see Figure S2), in which case the optimization converged to conformer NW3-1. It seems that the comparably small naphthalene substrate cannot easily accommodate an OH−π coordination to more than two water molecules. Also note that, unlike in NW3-2, in NW3-1 and NW3-3 the orientation of the water molecules in the cluster corresponds to that of the most stable configuration of the free water trimer.^24^ We initiated BOMD computations from the three configurations. The computations seeded with NW3-2 and NW3-3 relaxed after 20–30 ps to a configuration of the NW3-1 type, which then became dominant during the rest of the 100 ps trajectory. Hence, the different computational approaches consistently suggest that the main contributions to experimental observations must originate from configurations of the NW3-1 type. Figure 4 shows that this expectation is corroborated by the agreement of the BOMD spectrum computed for the NW3-1 conformation with the experiment in terms of band progression and widths, although sizable shifts for some of the bands exist, which could not be corrected with the single scaling factor (0.98) applied to the BOMD frequencies. Figure 4 shows that the NW3-1 configuration is comparably rigid, as the mobility of the trimer is restricted by the three-fold anchoring of the trimer to the naphthalene substrate (two OH−π bonds and one CH···O bond) and the internal H-bonding in the trimer. It can be noted that the positions of the O atoms of the water molecules vary by no more than 1 Å during the full 100 ps BOMD trajectory. Such rigidity would explain the similarity in the band widths of the water vibrational modes and of the pure naphthalene mode. Minor contributions from dynamically unstable conformations (e.g., of the NW3-2 and NW3-3 types) are uncertain but may add to the broad-band background observed in different regions of the experimental IR spectrum.

The fluxional character of the water–naphthalene complexes exposed in the present study is likely to constitute a general feature of the complexes of water with aromatic substrates. The main hydration motifs derived from this work for the NW1–NW3 complexes are coincident with those obtained in a previous spectroscopic investigation in the O-H stretching region, backed up by MP2 computations.^15^ Similar or closely related configurations have been detected for microhydrated anthracene,^23^ acenaphthene,^16,17^ and phenanthrene.^20^ The focus here is on the dynamic behavior of the water and water clusters and its effect on the spectral signatures of the complexes. Significant band broadenings and apparent anharmonicities, indicative of fluxional behavior, were observed in previous far-IR experiments on acenaphthene–water_1–3_ complexes.^16^ The band positions in the acenaphthene–water complexes differ from the ones observed here for naphthalene, presumably due to the additional out-of-plane aliphatic hydrogens of acenaphthene, which provide additional anchoring sites for the water molecules, also leading to more rigid complexes, in particular for the W1 case. The relevance of water diffusion on the aromatic framework of phenanthrene has been highlighted by high-resolution rotational spectroscopy experiments.^20^ Evidence for quantum tunneling of water along the ring structure of phenanthrene was provided in the form of spectral splittings, demonstrating active water dynamics that were rationalized based on isotopic substitution experiments and computations of the potential energy surface relevant for water motion. Molecular Dynamics computations for microhydrated anthracene, acenaphthene, phenanthrene, and related systems can be expected to provide insights into the dynamics of the (micro)hydrating environment of these benchmark aromatics.

Noticeable structural similarities are observed between the water clusters complexed to the aromatic substrate and the corresponding pure water clusters.^14,18^ In particular, the similarity of the W2 and W3 complexes is in line with other PAH–water studies, where a common conclusion is that the interaction between the water molecules dominates over water–PAH interactions.^12,13,17,56^ Incidentally, the present conclusion about the NW3 complex being more rigid than the NW1–2 complexes is somewhat contrary to the trend postulated by Molina and co-workers, who expect multiple conformers of larger PAH–water clusters.^23^ The observation of W2 far-IR signatures is valuable in the context of the earth’s atmosphere, and broadening of vibrational bands has been observed in pure water clusters associated with dynamics and coupling to the dissociation coordinate.^53^ A number of high-resolution gas phase far-IR spectra were recorded by the Saykally group, focusing on vibrational bands below 150 cm^–1^ and around 524 cm^–1^, which enabled them to disentangle vibration rotation tunneling splitting.^57^ More recently, Schwan and co-workers recorded the water dimer spectrum in a wider frequency range using the free-electron-laser FELIX.^25^ In their experiments, however, it is complex to disentangle the water dimer features from larger water clusters. Comparison of these latter measurements for W2 with the present ones for NW2 shows a resemblance in some spectral features. Namely, the bands for W2 around 130, 185, and 283 cm^–1^ may be cautiously associated with the ones presently observed for NW2 at 128, 176, and 252 cm^–1^. Several of the far-IR peaks in the NW3 spectrum are expected to originate from the modes of W3 itself. As was the case for NW2, the relatively small perturbation from naphthalene warrants comparison with pure W3 spectra. Cautiously, we could relate our experimental peaks at 189 and 252 cm^–1^ to the hydrogen bond asymmetric and symmetric stretches, respectively, calculated to be around 170–180 and 207–235 cm^–1^.^24,58^ The water libration, of which four bands have been observed between 515 and 528 cm^–1^, could be explained by the broad feature that we observe around 519 cm^–1^.^59,60^ The broader features at 601 or 699 cm^–1^ could correspond to the out-of-plane vibration.^24^ A similar comparison of both NW2 and NW3 can be made to the matrix isolation experiments reported by Ceponkus *et al.* for W2 and W3.^61^ Their dimer features around 311 and 525 cm^–1^ (in Ne) are relatively close to our experimental values at 252 and 479 cm^–1^ (the 479 cm^–1^ feature likely consists of both a naphthalene peak and a W2 feature in our experiment; see above). A comparable number of peaks are observed in the matrix isolation spectrum for W3 in the 200–600 cm^–1^ range as in the gas phase NW3 spectrum reported here, of which most above 310 cm^–1^ are assigned to combination bands. The presence of combination bands is in line with our conclusions about the significant effects of anharmonicity on the far-IR spectra of the NW1–3 complexes. It should be noted that the cold matrix likely influences the band frequencies, intensities, and overall mobility of the W cluster.

Far-IR action spectra directly probing intermolecular vibrations of naphthalene–water_1–3_ complexes are interpreted based on BOMD simulations. The spectra display features attributed to the mobility of water with respect to the PAH substrate. At the low but finite temperature in our molecular beam, the water monomer has a significant degree of freedom over the PAH surface, which reduces with increasing size of the clusters, owing to their ability to coordinate more strongly to naphthalene. Even though the interactions between water and naphthalene are relatively weak, they can affect the dynamics within the water dimer, as shown by our MD simulations. A comparison of harmonic and anharmonic calculations shows the dominating effects of the anharmonicity, especially for the larger water clusters. The presented size- and REMPI-selected far-IR action spectra together with BOMD simulations provide a reliable formalism for investigating weak bond formations between water and hydrophobic surfaces dominated by shallow potential energy surfaces.

## Methods

*Experimental.* Experiments were performed at the HFML-FELIX laboratory,^40^ where IR spectra were measured via IR-UV ion-dip spectroscopy in a molecular beam setup discussed in more detail previously.^41^ Naphthalene (99% purity) was heated in a glass sample compartment to 85 °C in front of a series 9 pulsed General Valve running at 20 Hz. Water was introduced by passing the backing gas (Ar, 6 bar) over an external reservoir filled with water (volume of ∼5 mL). The molecular clusters were probed using 1 + 1′ REMPI, with a Nd:YAG pumped dye laser and an ArF excimer laser aligned perpendicularly to the molecular beam. The resulting ions were mass-separated with a reflectron time-of-flight mass spectrometer. IR laser pulses were provided by the free-electron-laser FELIX, which is counter propagated with the molecular beam and precedes the ionization lasers by about 300 μs. FELIX was scanned between 100 and 900 cm^–1^, in steps of 1 cm^–1^. Figure S6 displays the power as a function of wavenumber. The IR laser was operated at a repetition rate of 10 Hz, allowing for acquiring alternating IR on and off ion mass spectra and correcting for fluctuations in ion signal. We notice that the current measurements present a significantly improved S/N ratio in comparison with previous experiments on microhydrated aromatics^16^ with the same setup, due to an improved collinear arrangement of the molecular and laser beams, resulting in a more efficient use of the available far-IR photons. Moreover, the well-separated UV transitions of the NW1–3 complexes (see below) largely reduce contributions of masses other than the targeted one to the IR spectrum through fragmentation.

*Computational.* A conformational search was performed for each cluster using the standard production run in CREST,^42^ from which complexes below 10 kJ/mol were considered for further optimization. Static DFT and MP2 geometry optimizations and frequency calculations were performed with the Gaussian16^43^ suite of programs. The 6-311++G** basis set was employed in all cases. For DFT, different functionals were tested, namely B3LYP-D3^44^ (with the GD3BJ empirical dispersion correction^45^), along with a variety of hybrid functionals differing in the treatment of long-range exchange-correlation interactions, namely M06-2x^46^ and wB97xD.^47^ Anharmonic spectra were calculated using GVPT2 as incorporated into Gaussian16, with the Fermi resonance threshold set to 100 cm^–1^ and the Darling–Dennison resonance threshold set to 50 cm^–1^.

BOMD calculations were performed with the CP2K package,^48^ using the B3LYP functional with the GD3BJ dispersion correction, the DZVP-GTH basis set, and Goedecker, Teter, and Hutter pseudopotentials.^49^ The cutoff radius for the pair potential was set to 12.5 Å, and a cubic cell of side length 25 Å was employed for the isolated complexes. The species were equilibrated for 3.5 ps in the NVT ensemble at 50 K, with the CSVR thermostat (canonical sampling through velocity rescaling), combining stages of GLOBAL (2.5 ps), and MASSIVE (1 ps) treatment of the internal degrees of freedom.^48^ We found that a simulation at an effective temperature of 50 K gives qualitatively good agreement in terms of spectral peak widths across the different cluster sizes. This temperature is in line with previous BOMD studies of biomolecules under similar molecular beam conditions.^36^ Since BOMD ignores tunneling and zero-point energy effects that can increase mobility, this is an effective temperature to simulate the mobility, resulting in band broadenings, but as such does not reflect the physical temperature. Subsequently, a production run was performed in the NVE ensemble to monitor the dynamics of the complexes over up to 100 ps, during which the temperature fluctuated with a standard deviation of 7 K. Such long BOMD trajectories sought an efficient sampling of the configurations of the water moiety around the naphthalene substrate, eventually leading to dynamic connections between different hydration conformations, although complete ergodicity is not ensured. Computational infrared spectra were produced with the TRAVIS analyzer package,^50^ from the Fourier transform of the time correlation function of the fluctuating dipole moment, as computed from the Wannier center coordinates produced during the BOMD trajectories.

For comparison with experiment, the frequencies of the computational spectra were scaled with the following factors: 0.98 for B3LYP and BOMD, 0.965 for M062x and wB97xD, and 1.01 for MP2. These factors were chosen to match the position of the intense and sharp naphthalene band detected at 786 cm^–1^ for the three complexes NW1–NW3.

## Supporting Information Available

A mass spectrum, an overview of lowest energy conformers, PESs of NW1 and NW2, IR spectra based on static DFT calculations, an overview of fitting parameters of experimental IR bands, and power of the IR free electron laser as used in this study. (PDF)
Transparent Peer Review report available (PDF)

## References

[ref1] Sacchi M.; Tamtögl A. Water Adsorption and Dynamics on Graphene and Other 2D Materials: Computational and Experimental Advances. Adv. Phys. X 2023, 8 (1), 1–42. 10.1080/23746149.2022.2134051. PMC7614201 36816858

[ref2] Wu M.; Wei W.; Liu X.; Liu K.; Li S. Structure and Dynamic Properties of Stretched Water in Graphene Nanochannels by Molecular Dynamics Simulation: Effects of Stretching Extent. Phys. Chem. Chem. Phys. 2019, 21 (35), 19163–19171. 10.1039/C9CP03981C. 31433424

[ref3] Martinez C. R.; Iverson B. L. Rethinking the Term “Pi-Stacking.”. Chem. Sci. 2012, 3 (7), 2191–2201. 10.1039/c2sc20045g.

[ref4] Schwing K.; Gerhards M. Investigations on Isolated Peptides by Combined IR/UV Spectroscopy in a Molecular Beam – Structure, Aggregation, Solvation and Molecular Recognition. Int. Rev. Phys. Chem. 2016, 35 (4), 569–677. 10.1080/0144235X.2016.1229331.

[ref5] Bakels S.; Meijer E. M.; Greuell M.; Porskamp S. B. A.; Rouwhorst G.; Mahé J.; Gaigeot M. P.; Rijs A. M. Interactions of Aggregating Peptides Probed by IR-UV Action Spectroscopy. Faraday Discuss. 2019, 217, 322–341. 10.1039/C8FD00208H. 31066731

[ref6] Pfeilsticker K.; Lotter A.; Peters C.; Bösch H. Atmospheric Detection of Water Dimers via Near-Infrared Absorption. Science 2003, 300 (5628), 2078–2080. 10.1126/science.1082282. 12829778

[ref7] Tretyakov M. Y.; Serov E. A.; Koshelev M. A.; Parshin V. V.; Krupnov A. F. Water Dimer Rotationally Resolved Millimeter-Wave Spectrum Observation at Room Temperature. Phys. Rev. Lett. 2013, 110 (9), 1–4. 10.1103/PhysRevLett.110.093001. 23496706

[ref8] Kulmala M. How Particles Nucleate and Grow. Science 2003, 302 (5647), 1000–1001. 10.1126/science.1090848. 14605359

[ref9] Tielens A. G. G. M. Molecular Astrophysics; Cambridge University Press: Cambridge, 2021.

[ref10] Lemmens A. K.; Rap D. B.; Brünken S.; Buma W. J.; Rijs A. M. Polycyclic Aromatic Hydrocarbon Growth in a Benzene Discharge Explored by IR-UV Action Spectroscopy. Phys. Chem. Chem. Phys. 2022, 24 (24), 14816–14824. 10.1039/D2CP01631A. 35695165 PMC9215700

[ref11] Andersen J.; Larsen R. W.; Ceponkus J.; Uvdal P.; Nelander B. Far-Infrared Investigation of the Benzene-Water Complex: The Identification of Large-Amplitude Motion and Tunneling Pathways. J. Phys. Chem. A 2020, 124 (3), 513–519. 10.1021/acs.jpca.9b01497. 31815483

[ref12] Chatterjee K.; Dopfer O. Protonation of Naphthalene-(Water)n Nanoclusters: Intracluster Proton Transfer to Hydration Shell Revealed by Infrared Photodissociation Spectroscopy. J. Phys. Chem. A 2020, 124 (6), 1134–1151. 10.1021/acs.jpca.9b11779. 31939665

[ref13] Knurr B. J.; Adams C. L.; Weber J. M. Infrared Spectroscopy of Hydrated Naphthalene Cluster Anions. J. Chem. Phys. 2012, 137 (10), 104303 10.1063/1.4750371. 22979855

[ref14] Pribble R. N.; Zwier T. S. Probing Hydrogen Bonding in Benzene-(Water)_n_ Clusters Using Resonant Ion-Dip IR Spectroscopy. Faraday Discuss. 1994, 97, 229–241. 10.1039/FD9949700229.

[ref15] Chatterjee K.; Roy T. K.; Khatri J.; Schwaab G.; Havenith M. Unravelling the Microhydration Frameworks of Prototype PAH by Infrared Spectroscopy: Naphthalene-(Water)1–3. Phys. Chem. Chem. Phys. 2021, 23 (25), 14016–14026. 10.1039/D1CP01789F. 34151322

[ref16] Lemmens A. K.; Gruet S.; Steber A. L.; Antony J.; Grimme S.; Schnell M.; Rijs A. M. Far-IR and UV Spectral Signatures of Controlled Complexation and Microhydration of the Polycyclic Aromatic Hydrocarbon Acenaphthene. Phys. Chem. Chem. Phys. 2019, 21, 3414–3422. 10.1039/C8CP04480E. 30378601

[ref17] Steber A. L.; Pérez C.; Temelso B.; Shields G. C.; Rijs A. M.; Pate B. H.; Kisiel Z.; Schnell M. Capturing the Elusive Water Trimer from the Stepwise Growth of Water on the Surface of the Polycyclic Aromatic Hydrocarbon Acenaphthene. J. Phys. Chem. Letters 2017, 8, 5744–5750. 10.1021/acs.jpclett.7b02695. 29112436

[ref18] Pribble R. N.; Zwier T. S. Size-Specific Infrared Spectra of Benzene-(H2O)n Clusters (n = 1 through 7): Evidence for Noncyclic (H2O)n Structures. Science 1994, 265 (5168), 75–79. 10.1126/science.265.5168.75. 17774690

[ref19] Pérez C.; Steber A. L.; Rijs A. M.; Temelso B.; Shields G. C.; Lopez J. C.; Kisiel Z.; Schnell M. Corannulene and Its Complex with Water: A Tiny Cup of Water. Phys. Chem. Chem. Phys. 2017, 19 (22), 14214–14223. 10.1039/C7CP01506B. 28474023

[ref20] Loru D.; Steber A. L.; Obenchain D. A.; Temelso B.; Lopez J. C.; Schnell M. Quantum Tunneling Facilitates Water Motion across the Surface of Phenanthrene. J. Am. Chem. Soc. 2023, 145 (31), 17201–17210. 10.1021/jacs.3c04281. 37494139 PMC10416304

[ref21] Loru D.; Steber A. L.; Pinacho P.; Gruet S.; Temelso B.; Rijs A. M.; Pérez C.; Schnell M. How Does the Composition of a PAH Influence Its Microsolvation? A Rotational Spectroscopy Study of the Phenanthrene-Water and Phenanthridine-Water Clusters. Phys. Chem. Chem. Phys. 2021, 23 (16), 9721–9732. 10.1039/D1CP00898F. 33870387

[ref22] Xu B.; Stein T.; Ablikim U.; Jiang L.; Hendrix J.; Head-Gordon M.; Ahmed M. Probing Solvation and Reactivity in Ionized Polycyclic Aromatic Hydrocarbon-Water Clusters with Photoionization Mass Spectrometry and Electronic Structure Calculations. Faraday Discuss. 2019, 217, 414–433. 10.1039/C8FD00229K. 31016308

[ref23] Rossich Molina E.; Xu B.; Kostko O.; Ahmed M.; Stein T. A Combined Theoretical and Experimental Study of Small Anthracene-Water Clusters. Phys. Chem. Chem. Phys. 2022, 24 (38), 23106–23118. 10.1039/D2CP02617A. 35975620

[ref24] Keutsch F. N.; Cruzan J. D.; Saykally R. J. The Water Trimer. Chem. Rev. 2003, 103 (7), 2533–2577. 10.1021/cr980125a. 12848579

[ref25] Schwan R.; Qu C.; Mani D.; Pal N.; van der Meer L.; Redlich B.; Leforestier C.; Bowman J. M.; Schwaab G.; Havenith M. Observation of the Low-Frequency Spectrum of the Water Dimer as a Sensitive Test of the Water Dimer Potential and Dipole Moment Surfaces. Angew. Chem., Int. Ed. 2019, 58 (37), 13119–13126. 10.1002/anie.201906048. PMC7687217 31350942

[ref26] Vaida V. Perspective: Water Cluster Mediated Atmospheric Chemistry. J. Chem. Phys. 2011, 135 ( (2), ), 020901 10.1063/1.3608919. 21766916

[ref27] Daniel J. S.; Solomon S.; Kjaergaard H. G.; Schofield D. P. Atmospheric Water Vapor Complexes and the Continuum. Geophys. Res. Lett. 2004, 31 (6), 1–4. 10.1029/2003GL018914.

[ref28] Chatterjee K.; Dopfer O. Infrared Spectroscopy of Hydrated Polycyclic Aromatic Hydrocarbon Cations: Naphthalene+-Water. Phys. Chem. Chem. Phys. 2017, 19 (48), 32262–32271. 10.1039/C7CP06893J. 29192906

[ref29] Chatterjee K.; Dopfer O. Microhydration of PAH+ Cations: Evolution of Hydration Network in Naphthalene-(H2O)_n_ Clusters (*n* ≤ 5). Chem. Sci. 2018, 9 (8), 2301–2318. 10.1039/C7SC05124G. 29719704 PMC5903421

[ref30] Chatterjee K.; Dopfer O. Infrared Spectroscopy of Hydrated Polycyclic Aromatic Hydrocarbon Cations: Naphthalene^+^ – Water. Phys. Chem. Chem. Phys. 2017, 19 (48), 32262–32271. 10.1039/C7CP06893J. 29192906

[ref31] Alata I.; Broquier M.; Dedonder-Lardeux C.; Jouvet C.; Kim M.; Sohn W. Y.; Kim S. S.; Kang H.; Schtz M.; Patzer A.; Dopfer O. Microhydration Effects on the Electronic Spectra of Protonated Polycyclic Aromatic Hydrocarbons: [Naphthalene-(H2O)_n_1,2]H. J. Chem. Phys. 2011, 134 ( (7), ), 074307 10.1063/1.3554416. 21341844

[ref32] Sharma D.; Paterson M. J. Ground and Excited States of Naphthalene-Water (Naphtha-W6) Clusters: A Computational Study. RSC Adv. 2015, 5 (36), 28281–28291. 10.1039/C5RA01894C.

[ref33] Lemmens A. K.; Rap D. B.; Thunnissen J. M. M.; Gruet S.; Steber A. L.; Panchagnula S.; Tielens A. G. G. M.; Schnell M.; Buma W. J.; Rijs A. M. Far-IR Absorption of Neutral Polycyclic Aromatic Hydrocarbons (PAHs): Light on the Mechanism of IR – UV Ion Dip Spectroscopy ´. J. Phys. Chem. Lett. 2020, 11, 8997–9002. 10.1021/acs.jpclett.0c02714. 33035060 PMC7649846

[ref34] Lemmens A. K.; Rijs A. M.; Buma W. J. Infrared Spectroscopy of Jet-Cooled “GrandPAHs” in the 3–100 Mm Region. Astrophys. J. 2021, 923 (2), 238 10.3847/1538-4357/ac2f9d.

[ref35] Cabaleiro-Lago E. M.; Rodriguez-Otero J.; Pena-Gallego A. Computational Study on the Characteristics of the Interaction in Naphthalene ···(H_2_X)_n_)_1,2_ (X) O, S) Clusters. J. Phys. Chem. A 2008, 112, 6344–6350. 10.1021/jp8021979. 18570360

[ref36] Mahé J.; Jaeqx S.; Rijs A. M.; Gaigeot M.-P. Can Far-IR Action Spectroscopy Combined with BOMD Simulations Be Conformation Selective?. Phys. Chem. Chem. Phys. 2015, 17, 25905–25914. 10.1039/C5CP01518A. 26054490

[ref37] Lambros E.; Paesani F. How Good Are Polarizable and Flexible Models for Water: Insights from a Many-Body Perspective. J. Chem. Phys. 2020, 153 ( (6), ), 060901 10.1063/5.0017590. 35287447

[ref38] Jaeqx S.; Oomens J.; Cimas A.; Gaigeot M. P.; Rijs A. M. Gas-Phase Peptide Structures Unraveled by Far-IR Spectroscopy: Combining IR-UV Ion-Dip Experiments with Born-Oppenheimer Molecular Dynamics Simulations. Angew. Chem., Int. Ed. 2014, 53 (14), 3663–3666. 10.1002/anie.201311189. 24574197

[ref39] Belega E. D.; Zakirov M. N.; Chulichkov A. I.; Trubnikov D. N.; Novakovskaya Yu. V. Effective-Mode Analysis of the Dynamics of Weakly Bound Molecular Systems by an Example of Hydrogen-Bonded Water Clusters. Phys. Rev. A. 2023, 107 (3), 1–15. 10.1103/PhysRevA.107.032812.

[ref40] Oepts D.; van der Meer A.F.G.; van Amersfoort P.W. The Free-Electron-Laser User Facility FELIX. Infrared Phys. Technol. 1995, 36, 297–308. 10.1016/1350-4495(94)00074-U.

[ref41] Rijs A. M.; Kabeláč M.; Abo-Riziq A.; Hobza P.; De Vries M. S. Isolated Gramicidin Peptides Probed by IR Spectroscopy. ChemPhysChem 2011, 12 (10), 1816–1821. 10.1002/cphc.201100212. 21656894

[ref42] Pracht P.; Bohle F.; Grimme S. Automated Exploration of the Low-Energy Chemical Space with Fast Quantum Chemical Methods. Phys. Chem. Chem. Phys. 2020, 22 (14), 7169–7192. 10.1039/C9CP06869D. 32073075

[ref43] Frisch M. J.; Trucks G. W.; Schlegel H. B.; Scuseria G. E.; Robb M. A.; Cheeseman J. R.; Scalmani G.; Barone V.; Petersson G. A.; Nakatsuji H.; Li X.; Caricato M.; Marenich A. V.; Bloino J.; Janesko B. G.; Gomperts R.; Mennucci B.; Hratchian H. P.; Ortiz J. V.; Izmaylov A. F.; Sonnenberg J. L.; Williams-Young D.; Ding F.; Lipparini F.; Egidi F.; Goings J.; Peng B.; Petrone A.; Henderson T.; Ranasinghe D.; Zakrzewski V. G.; Gao J.; Rega N.; Zheng G.; Liang W.; Hada M.; Ehara M.; Toyota K.; Fukuda R.; Hasegawa J.; Ishida M.; Nakajima T.; Honda Y.; Kitao O.; Nakai H.; Vreven T.; Throssell K.; Montgomery J. A. Jr.; Peralta J. E.; Ogliaro F.; Bearpark M. J.; Heyd J. J.; Brothers E. N.; Kudin K. N.; Staroverov V. N.; Keith T. A.; Kobayashi R.; Normand J.; Raghavachari K.; Rendell A. P.; Burant J. C.; Iyengar S. S.; Tomasi J.; Cossi M.; Millam J. M.; Klene M.; Adamo C.; Cammi R.; Ochterski J. W.; Martin R. L.; Morokuma K.; Farkas O.; Foresman J. B.; Fox D. J. Gaussian 16, Rev. A.03; Gaussian, Inc.: Wallingford, CT, 2016.

[ref44] Becke A. D. Density-functional Thermochemistry. III. The Role of Exact Exchange. J. Chem. Phys. 1993, 98 (7), 5648–5652. 10.1063/1.464913.

[ref45] Grimme S.; Antony J.; Ehrlich S.; Krieg H. A Consistent and Accurate Ab Initio Parametrization of Density Functional Dispersion Correction (DFT-D) for the 94 Elements H-Pu. J. Chem. Phys. 2010, 132 (15), 154104 10.1063/1.3382344. 20423165

[ref46] Zhao Y.; Truhlar D. G. The M06 Suite of Density Functionals for Main Group Thermochemistry, Thermochemical Kinetics, Noncovalent Interactions, Excited States, and Transition Elements: Two New Functionals and Systematic Testing of Four M06-Class Functionals and 12 Other Function. Theor. Chem. Acc. 2008, 120 (1–3), 215–241. 10.1007/s00214-007-0310-x.

[ref47] Chai J. Da; Head-Gordon M. Long-Range Corrected Hybrid Density Functionals with Damped Atom-Atom Dispersion Corrections. Phys. Chem. Chem. Phys. 2008, 10 (44), 6615–6620. 10.1039/b810189b. 18989472

[ref48] Kühne T. D.; Iannuzzi M.; Del Ben M.; Rybkin V. V.; Seewald P.; Stein F.; Laino T.; Khaliullin R. Z.; Schütt O.; Schiffmann F.; Golze D.; Wilhelm J.; Chulkov S.; Bani-Hashemian M. H.; Weber V.; Borštnik U.; Taillefumier M.; Jakobovits A. S.; Lazzaro A.; Pabst H.; Müller T.; Schade R.; Guidon M.; Andermatt S.; Holmberg N.; Schenter G. K.; Hehn A.; Bussy A.; Belleflamme F.; Tabacchi G.; Glöß A.; Lass M.; Bethune I.; Mundy C. J.; Plessl C.; Watkins M.; VandeVondele J.; Krack M.; Hutter J. CP2K: An Electronic Structure and Molecular Dynamics Software Package -Quickstep: Efficient and Accurate Electronic Structure Calculations. J. Chem. Phys. 2020, 152 ( (19), ), 194103 10.1063/5.0007045. 33687235

[ref49] Goedecker S.; Teter M.; Hutter J. Separable Dual-Space Gaussian Pseudopotentials. Phys Rev B Condens Matter. Mater. Phys. 1996, 54 (3), 1703–1710. 10.1103/PhysRevB.54.1703. 9986014

[ref50] Brehm M.; Thomas M.; Gehrke S.; Kirchner B. TRAVIS—A Free Analyzer for Trajectories from Molecular Simulation. J. Chem. Phys. 2020, 152 ( (16), ), 164105 10.1063/5.0005078. 32357781

[ref51] Beck S. M.; Powers D. E.; Hopkins J. B.; Smalley R. E. Jet-Cooled Naphthalene. I. Absorption Spectra and Line Profiles. J. Chem. Phys. 1980, 73 (5), 2019–2028. 10.1063/1.440421.

[ref52] Lemmens A. K.; Rap D. B.; Thunnissen J. M. M.; Mackie C. J.; Candian A.; Tielens A. G. G. M.; Rijs A. M.; Buma W. J. Anharmonicity in the Mid-Infrared Spectra of Polycyclic Aromatic Hydrocarbons. Molecular Beam Spectroscopy and Calculations. Astron. Astrophys. 2019, 628, A130 10.1051/0004-6361/201935631.

[ref53] Paul J. B.; Provencal C.; Chapo C.; Roth K.; Casaes R.; Saykally R. J. Infrared Cavity Ringdown Spectroscopy of the Water Cluster Bending Vibrations. J. Phys. Chem. A 1999, 103 (16), 2972–2974. 10.1021/jp984618v.

[ref54] Ghosh S. R.; Debnath B.; Jana A. D. Water Dimer Isomers: Interaction Energies and Electronic Structure. J. Mol. Model. 2020, 26 (1), 1–9. 10.1007/s00894-019-4274-2. 31907630

[ref55] Fellers R. S.; Leforestier C.; Braly L. B.; Brown M. C.; Saykally R. J. Spectroscopic Determination of the Water Pair Potential. Science 1999, 284 (5416), 945–948. 10.1126/science.284.5416.945. 10320371

[ref56] Palmer P. M.; Chen Y.; Topp M. R. Perylene/Water Clusters: Some Different Trends in Hydrogen-Bonded Structure Induced by a Large Aromatic Template. Chem. Phys. Lett. 2000, 325 (5–6), 568–576. 10.1016/S0009-2614(00)00731-4.

[ref57] Cruzan J. D.; Braly L. B.; Liu K.; Brown M. G.; Loeser J. G.; Saykally R. J. Quantifying Hydrogen Bond Cooperativity in Water: VRT Spectroscopy of the Water Tetramer. Science 1996, 271 (5245), 59–62. 10.1126/science.271.5245.59. 11536731

[ref58] Xantheas S. S.; Dunning T. H. Ab Initio Studies of Cyclic Water Clusters (H2O)n, N = 1–6. I. Optimal Structures and Vibrational Spectra. J. Chem. Phys. 1993, 99 (11), 8774–8792. 10.1063/1.465599.

[ref59] Cole W. T. S.; Fellers R. S.; Viant M. R.; Leforestier C.; Saykally R. J. Far-Infrared VRT Spectroscopy of the Water Dimer: Characterization of the 20 μ m out-of-Plane Librational Vibration. J. Chem. Phys. 2015, 143 ( (15), ), 154306 10.1063/1.4933116. 26493906

[ref60] Keutsch F. N.; Fellers R. S.; Viant M. R.; Saykally R. J. Far-Infrared Laser Vibration-Rotation-Tunneling Spectroscopy of Water Clusters in the Librational Band Region of Liquid Water. J. Chem. Phys. 2001, 114 (9), 4005–4015. 10.1063/1.1337052.

[ref61] Ceponkus J.; Karlström G.; Nelander B. Intermolecular Vibrations of the Water Trimer, a Matrix Isolation Study. J. Phys. Chem. A 2005, 109 (35), 7859–7864. 10.1021/jp052096v. 16834166

