# Supplementary material for: Wetting of a Hydrophobic Surface: Far-IR Action Spectroscopy and Dynamics of Microhydrated Naphthalene
Source: J Phys Chem Lett. 2023 Nov 28;14(48):10794–802. doi: 10.1021/acs.jpclett.3c02854 (PMC10711790; doi:10.1021/acs.jpclett.3c02854)
Supplement: Supplementary file 1 — jz3c02854_si_001.pdf [file jz3c02854_si_001.pdf]

Supporting Information

Wetting of a Hydrophobic Surface: Far-IR Action Spectroscopy and Dynamics of Microhydrated Naphthalene

Alexander K. Lemmens[1,2], Piero Ferrari[2], Donatella Loru[3], Gayatri Batra[3], Amanda L. Steber[4], Britta Redlich[2], Melanie Schnell[3,5], Bruno Martinez-Haya[6]

[1] Chemical Science Division, Lawrence Berkeley National Laboratory, Berkeley, California 94720, United States
[2] Radboud University, Institute of Molecules and Materials, HFML-FELIX, Toernooiveld 7, 6525 ED Nijmegen, The Netherlands
[3] Deutsches Elektronen-Synchrotron DESY, Notkestr. 85, 22607 Hamburg, Germany
[4] Department of Physical and Inorganic Chemistry, Faculty of Science, University of Valladolid, 47011 Valladolid, Spain
[5] Institut für Physikalische Chemie, Christian-Albrechts-Universität zu Kiel, Max-Eyth-Str. 1, 24118 Kiel
[6] Center for Nanoscience and Sustainable Technologies (CNATS), Department of Physical, Chemical and Natural Systems, Universidad Pablo de Olavide, 41013 Seville, Spain

Corresponding Author

aklemmens@lbl.gov

Table of contents

Figure S1: TOF-MS spectra of naphthalene-water complexes

Figure S2: Overview of lowest energy conformers predicted using different leveles of theory

Figure S3: Energy of the NW1 complex as function of different angular positions of water with respect to naphthalene

Figure S4: Single point energies along an excerpt of the BOMD trajectory where the NW2-2 transitions to the NW2-1

Figure S5: Comparison of experimental far-IR spectra to different predicted IR spectra based on static quantum chemical calculations

Table S1: Overview of gaussian-fitted experimental far-IR peaks of the NW1-3 clusters.

Figure S6: Power of the IR Free electron laser FELIX as function of wavelength as used in the experiments reported in this study

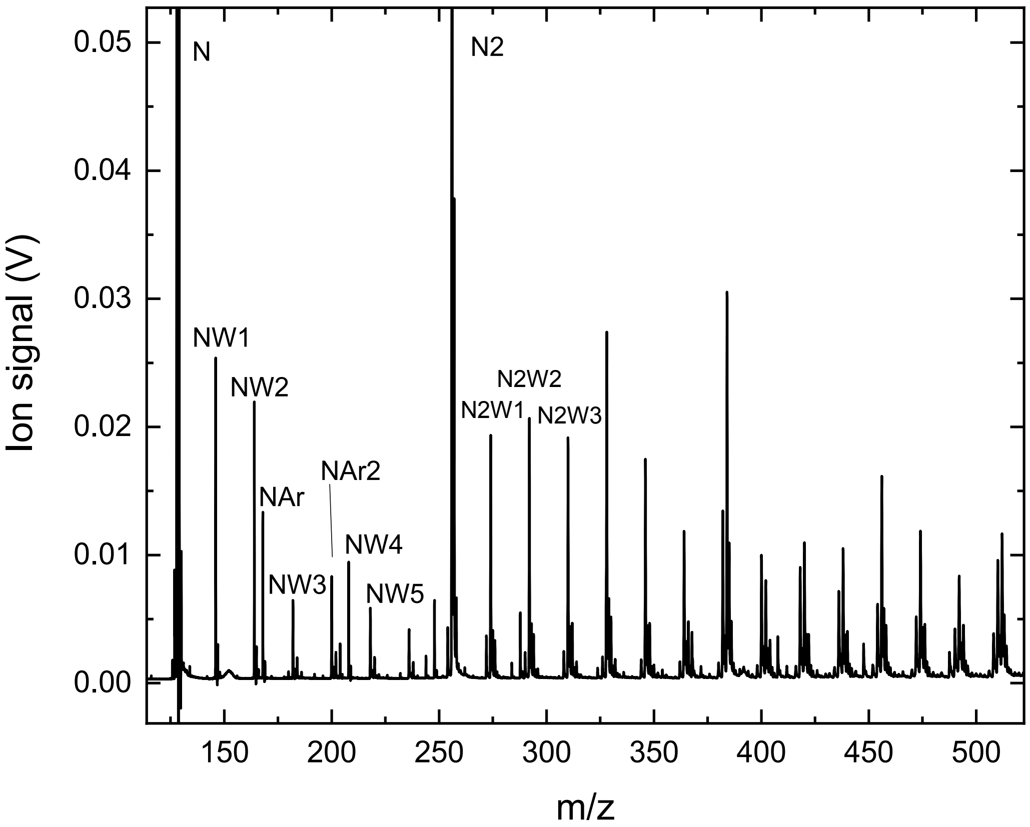

Figure S1: Calibrated TOF mass spectra of naphthalene (nap) complexes with water. Microhydration of nap with multiple water molecules is readily achieved. The trace is recorded at the first electronic transition of the nap-water complex at 32432 cm^{-1} with ionization by a 193 nm excimer laser pulse. The y-axis is truncated to increase the visibility of the clusters. N=0.95 N2=0.19 V

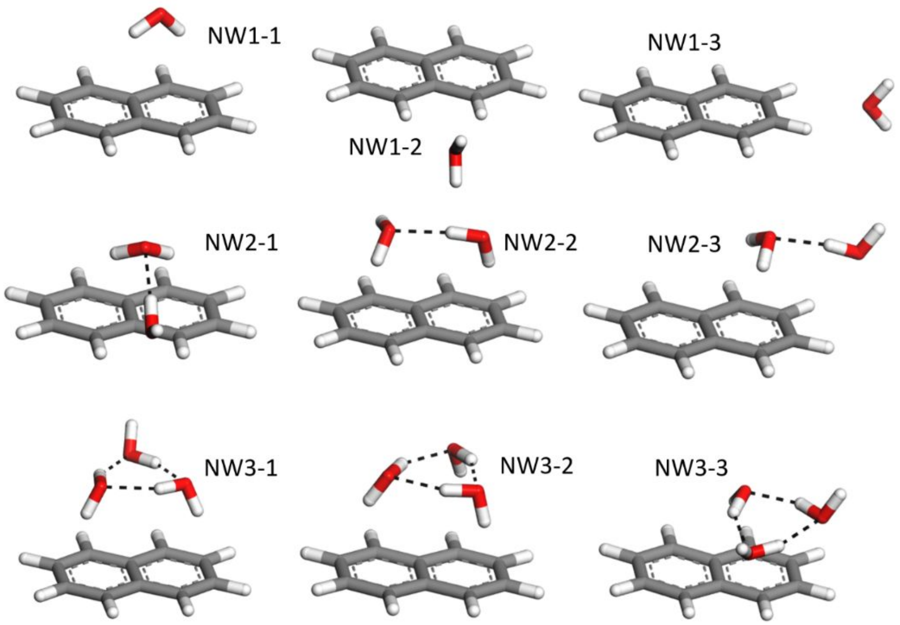

	**B3LYP 6-311++G****	**M06-2x 6-311++G****	**wB97xD 6-311++G****	**MP2 6-311++G****
**NW1-1**	0.0 (0.0)	0.0 (0.0)	0.0 (0.0)	0.0 (0.0)
**NW1-2**	+8.7 (+10.1)	+11.3 (+13.8)	+9.8 (+11.4)	+12.2 (+16.5)
**NW1-3**	+10.7 (+10.4)	+12.6 (+14.3)	+11.8 (+11.1)	+14.5 (+17.2)
**NW2-1**	0.0 (0.0)	0.0 (0.0)	0.0 (0.0)	0.0 (0.0)
**NW2-2**	+2.0 (+0.5)	+5.0 (-3.5)	+2.4 (+0.4)	+4.3 (+3.6)
**NW2-3**	unstable, it relaxes to NW2-1 upon optimization			
**NW3-1**	0.0 (0.0)	0.0 (0.0)	0.0 (0.0)	0.0 (0.0)
**NW3-2**	+5.1 (+1.4)	relaxes to NW3-1	+5.0 (+1.9)	relaxes to NW3-1
**NW3-3**	+7.9 (+1.6)	+9.5 (+6.7)	+7.8 (+1.8)	+7.9 (+2.7)

Figure S2: Lowest energy conformations of the NW1-3 hydrated naphthalene complexes. Relative zero point corrected electronic energies (Free energies in parenthesis) are indicated in kJ/mol at several levels of theory; namely MP2 and DFT with the indicated functionals and basis set.

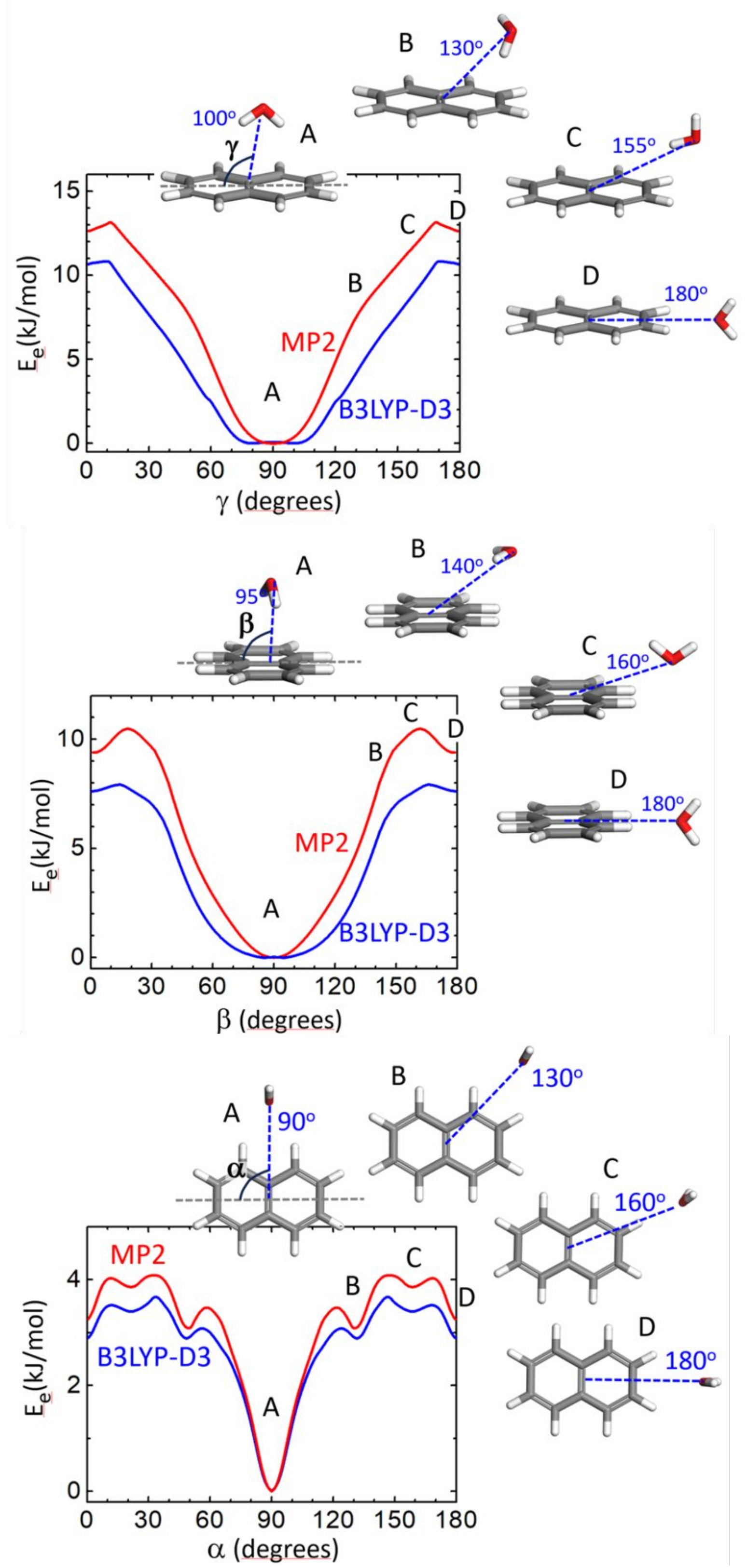

Figure S3: Energy of the NW1 complex as function of different angular positions of water with respect to naphthalene as indicated in the structures A-D calculated using two levels of theory as indicated in the plots.

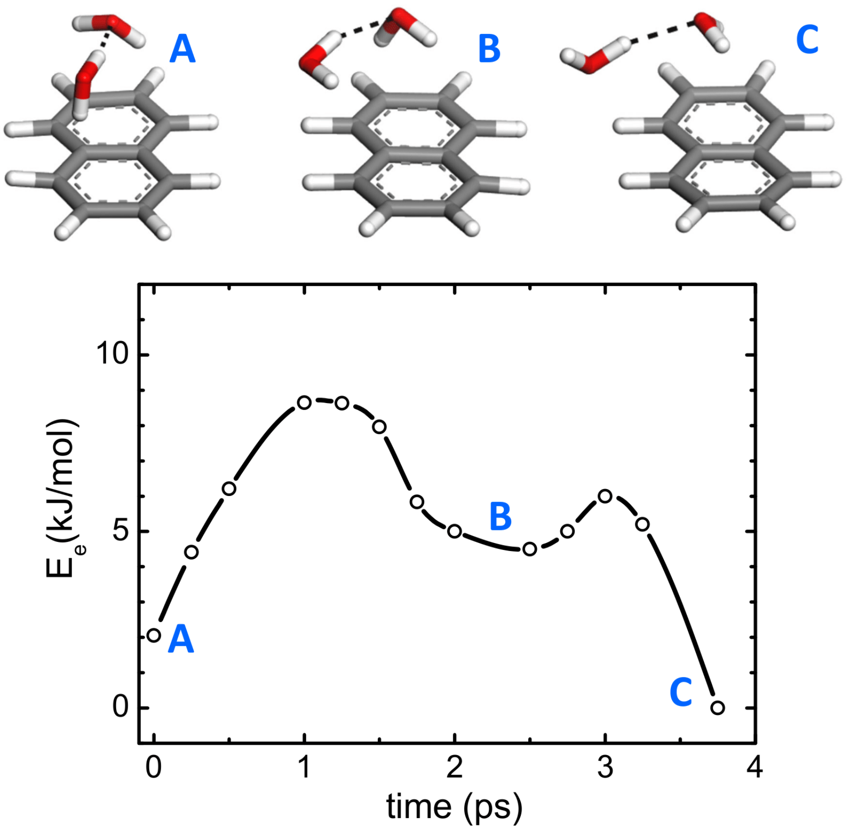

*Figure S4: Electronic energies (B3LYP/6-311++G**) for a set of points along a transit from the BOMD dynamics of NW2 in which the system migrates from NW2-2 to NW2-1, illustrating the barrier that is surmounted during the dynamics. Note that the initial (A) and end (C) configurations resemble NW2-2 and NW2-1, respectively, but are slightly different than the DFT optimized structures. There is a local minimum and barrier midway related to the required rotation of the water molecule and its diffusion to the naphthalene rim.*

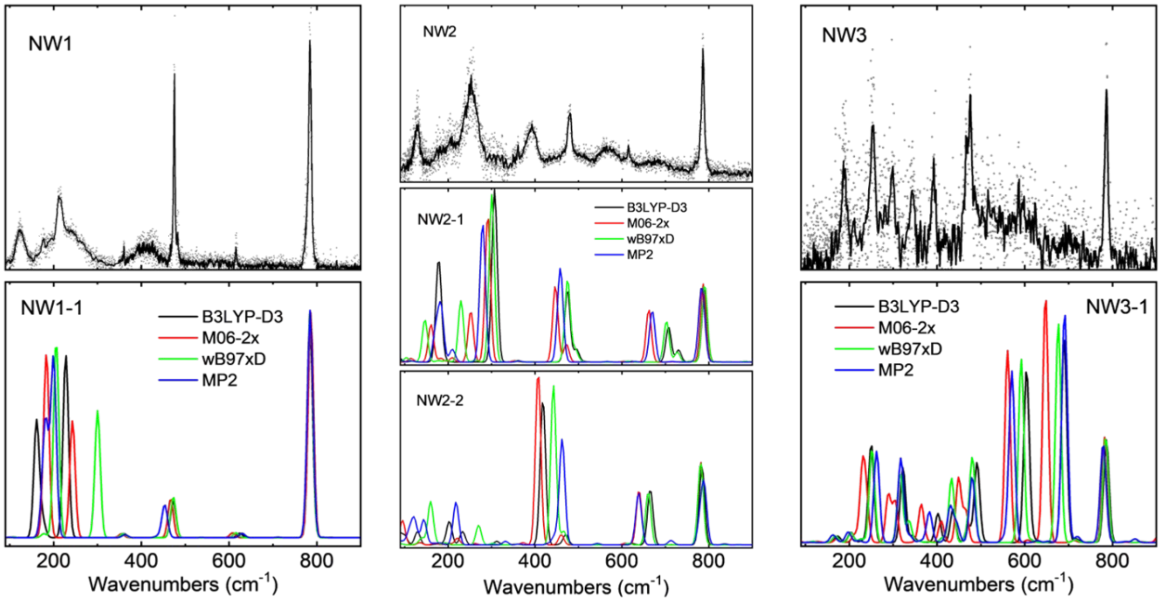

Figure S5: Far-IR gas phase IR-UV ion dip action spectra of jet-cooled NW1-3 complexes in black, an average of typically three IR scans of 30 averages per wavenumber (grey dots). In color are static simulated IR spectra (scaling factor in Methods section) for the lowest energy NW conformers.

Table S1: Gaussian-fitted experimental far-IR peaks of the NW1-3 clusters. The green numbers correspond to transitions involving the N substrate, black transitions are transitions that arise from the complexation with W. The orange fit is a background fit with an unclear origin.

	**Frequency (cm^{-1})**	**Gaussian width**	**area**
**NW1**	784	7.9	7.5
	615	3.5	0.25
	475	3.6	2.9
	422	102	7.1
	360	0.9	0.1
	225	75.8	13.2
	213	10.2	1.8
	176	5.5	0.3
	124	20.2	3.3
**NW2**	786	7.9	4.7
	683	25.1	0.5
	615	5.7	0.4
	570	33.6	2.3
	479	10.3	2.5
	410	682	103
	391	20.4	3.4
	361	2,4	0.2
	252	29.2	11.9

	206	26.5	2.9
	176	17.4	1.1
	128	13.6	2.9
**NW3**	786	9.9	3.2
	699	57.7	2
	601	44.7	2.5
	519	157.5	13.4
	473	16.8	3.7
	392	12.2	1.9
	342	17.8	2.2
	295	27.3	4.0
	252	23.9	5.4
	189	18.9	3.1

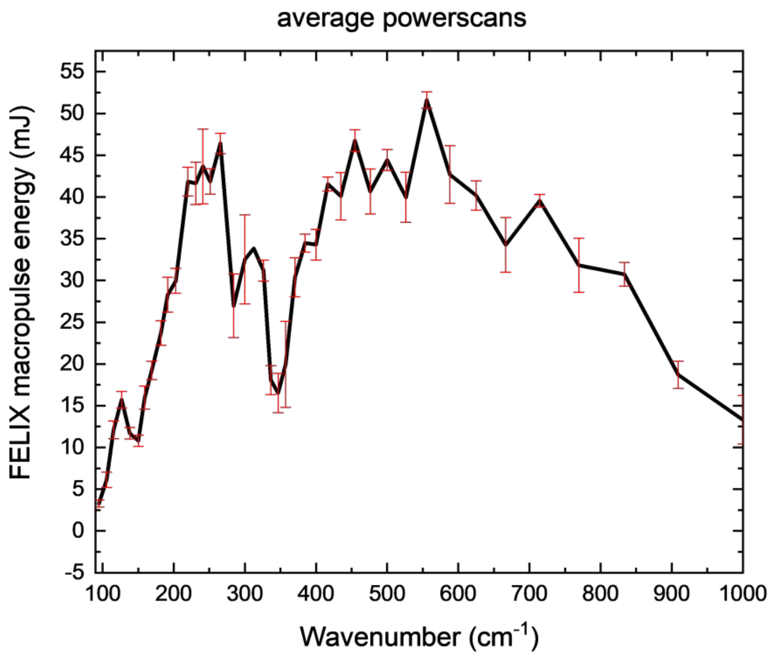

Figure S6: Power of the IR Free electron laser FELIX as function of wavelength as used in the experiments reported in this study.
