# Supplementary material for: Wetting of a Hydrophobic Surface: Far-IR Action Spectroscopy and Dynamics of Microhydrated Naphthalene
Source: J Phys Chem Lett. 2023 Nov 28;14(48):10794–802. doi: 10.1021/acs.jpclett.3c02854 (PMC10711790; doi:10.1021/acs.jpclett.3c02854)
Supplement: Supplementary file 2 — jz3c02854_si_002.pdf [file jz3c02854_si_002.pdf]

jz-2023-02854c.R1

Name: Peer Review Information for "Wetting of a Hydrophobic Surface: Far-IR Action Spectroscopy and Dynamics of Microhydrated Naphthalene"

First Round of Reviewer Comments

Reviewer: 1

Comments to the Author

In this work, Lemmens et al. report the UV-REMPI and far-IR spectra of neutral naphthalene-water clusters. They compare their experiments with computational work based on different approaches (static DFT with different functionals, MP2, BOMD). They find that dynamic effects are crucial to describe the signatures of the water-naphthalene interactions, as the water molecules explore the very shallow potential energy surface that characterizes the structures of these clusters.

This work on a very challenging problem showcases state-of-the-art experiments and computations. It will be of interest to the broad readership of JPCL, and it clearly deserves publication. I have a few comments that the authors should take into account before the paper is published:

1.) In Figure 2.e, the trajectory seems to be asymmetric, but there is no intrinsic reason for this asymmetry. Does this reflect a dependence of the structure on the starting point of the BOMD trajectories, or is this an artifact of a small number of trajectories (the authors do not mention the overall number of trajectories)?

2.) Can the authors report values for the temperature fluctuations in their BOMD simulations?

3.) The O-atom spatial distribution in the BOMD simulations seem to largely reflect the structural motifs from the static DFT calculations, although of course the dynamic effects giving rise to the line widths cannot be inferred from the static calculations. However, it would be interesting to have an idea of the probability distribution for finding the clusters in a given structural isomer. This distribution could be used to construct an IR spectrum from the superposition of the IR spectra of the different static isomers. However, this requires that the BOMD trajectories were sufficiently numerous and sufficiently long to explore the potential energy surface of each cluster ergodically. Is this the case here?

4.) For the water trimer complex, the isomers that seem to have been mainly explored are those with 2 or 3 pi-H-bonds. It would be interesting to have a probability distribution of the H-bonding configuration and a characteristic time scale for the switch. Since the BOMD simulations do not capture the quantum nature of the protons, but the work by, e.g., Saykally on the free water trimer clearly indicates the presence of tunneling, how much of an error in the dynamics is introduced by treating the protons classically?

Author's Response to Peer Review Comments:

Prof. Editor
The Journal of Physical Chemistry Letters

Lawrence Berkeley National Lab
1 Cyclotron Rd.
Berkeley
CA, 94708
Tel.: +19252556001
E-mail: aklemmens@lbl.gov

Berkeley, November 9, 2023

Dear Prof. Editor,

Thank you for considering our manuscript for publication in the Journal of Physical Chemistry Letters. We would also like to thank the reviewer for their positive feedback on our manuscript. We acknowledge the efforts of the reviewer in improving our manuscript by raising questions about the BOMD simulations that are central in interpreting the data. We will discuss the concerns and remarks in detail below, and we have highlighted significant changes in red in our manuscript. Additionally, we have made the suggested non-scientific changes in our revised manuscript.

1.) In Figure 2.e, the trajectory seems to be asymmetric, but there is no intrinsic reason for this asymmetry. Does this reflect a dependence of the structure on the starting point of the BOMD trajectories, or is this an artifact of a small number of trajectories (the authors do not mention the overall number of trajectories)?

We think the reviewer is right in saying that in the limit of a long simulation, the distribution of the oxygen over the naphthalene surface should reflect the symmetry of naphthalene. In order to preserve the information of the trajectory of NW1 in the paper, however, we do not symmetrize the plot. The asymmetry is now explained in the text, as shown below, as well as mentioning the length of the trajectory:

"The asymmetry of the spatial distribution in the current 100 ps long trajectory should disappear in the limit of long simulation times, noting, however, that the infrared spectrum is unlikely to change significantly with longer runs."

2.) Can the authors report values for the temperature fluctuations in their BOMD simulations?

The temperature fluctuation is now reported in the following sentence in the methods section:

"Subsequently, a production run was performed in the NVE ensemble to monitor the dynamics of the complexes over up to 100 ps, during which the temperature fluctuated with a standard deviation of 7 K."

3.) The O-atom spatial distribution in the BOMD simulations seem to largely reflect the structural motifs from the static DFT calculations, although of course the dynamic effects giving rise to the line widths cannot be inferred from the static calculations. However, it would be interesting to have an idea of the probability distribution for finding the clusters in a given structural isomer. This distribution could be used to construct an IR spectrum from the superposition of the IR spectra of the different static isomers. However, this requires that the BOMD trajectories were sufficiently numerous and sufficiently long to explore the potential energy surface of each cluster ergodically. Is this the case here?

The 2D trajectory plots already reflect the probability of different conformations. For the complex with the water dimer, where the BOMD simulations appear to connect two DFT minima, the suggested approach would be reasonable. However, much longer and more numerous BOMD trajectories are necessary to ensure ergodicity, which is now mentioned in the main text. Besides that, the static DFT computations are only used to qualitatively rationalize the observed IR spectra and form an incomplete description of this inherently dynamic system at finite temperatures that, as pointed out by the reviewer, results in band broadenings that are not accounted for by DFT.

4.) For the water trimer complex, the isomers that seem to have been mainly explored are those with 2 or 3 pi-H-bonds. It would be interesting to have a probability distribution of the H-bonding configuration and a characteristic time scale for the switch. Since the BOMD simulations do not capture the quantum nature of

the protons, but the work by, e.g., Saykally on the free water trimer clearly indicates the presence of tunneling, how much of an error in the dynamics is introduced by treating the protons classically?

We have considered all water trimer configurations, which as mentioned in the text ("The computations seeded with NW3-2 and NW3-3 relaxed after 20-30 ps to a configuration of the NW3-1 type, which then became dominant during the rest of the 100 ps trajectory."), convert to the NW3-1 type in 20-30 ps in the BOMD simulations. As the reviewer points out, tunneling effects are not captured by BOMD which results in us choosing an effective temperature that does not entirely reflect the physical temperature. Hence, we added to the text: "Since BOMD ignores tunneling and zero-point energy effects that can increase mobility, this is an effective temperature to simulate the mobility resulting in band broadenings, but as such does not reflect the physical temperature."

Hereby we believe to have addressed all concerns and comments and appreciate your efforts in considering our manuscript for publication in the Journal of Physical Chemistry Letters.

Sincerely yours and on behalf of all authors,

Alexander Lemmens
